# Forecasting Seizures in Dogs with Naturally Occurring Epilepsy

**DOI:** 10.1371/journal.pone.0081920

**Published:** 2014-01-08

**Authors:** J. Jeffry Howbert, Edward E. Patterson, S. Matt Stead, Ben Brinkmann, Vincent Vasoli, Daniel Crepeau, Charles H. Vite, Beverly Sturges, Vanessa Ruedebusch, Jaideep Mavoori, Kent Leyde, W. Douglas Sheffield, Brian Litt, Gregory A. Worrell

**Affiliations:** 1 NeuroVista Corp., Seattle, Washington, United States of America; 2 Veterinary Medical Center, University of Minnesota, St. Paul, Minnesota, United States of America; 3 Mayo Systems Electrophysiology Laboratory, Mayo Clinic, Rochester, Minnesota, United States of America; 4 School of Veterinary Medicine, University of Pennsylvania, Philadelphia, Pennsylvania, United States of America; 5 Veterinary School, University of California Davis, Davis, California, United States of America; 6 Department of Bioengineering, University of Pennsylvania, Philadelphia, Pennsylvania, United States of America; University of California, Riverside, United States of America

## Abstract

Seizure forecasting has the potential to create new therapeutic strategies for epilepsy, such as providing patient warnings and delivering preemptive therapy. Progress on seizure forecasting, however, has been hindered by lack of sufficient data to rigorously evaluate the hypothesis that seizures are preceded by physiological changes, and are not simply random events. We investigated seizure forecasting in three dogs with naturally occurring focal epilepsy implanted with a device recording continuous intracranial EEG (iEEG). The iEEG spectral power in six frequency bands: delta (0.1–4 Hz), theta (4–8 Hz), alpha (8–12 Hz), beta (12–30 Hz), low-gamma (30–70 Hz), and high-gamma (70–180 Hz), were used as features. Logistic regression classifiers were trained to discriminate labeled pre-ictal and inter-ictal data segments using combinations of the band spectral power features. Performance was assessed on separate test data sets via 10-fold cross-validation. A total of 125 spontaneous seizures were detected in continuous iEEG recordings spanning 6.5 to 15 months from 3 dogs. When considering all seizures, the seizure forecasting algorithm performed significantly better than a Poisson-model chance predictor constrained to have the same time in warning for all 3 dogs over a range of total warning times. Seizure clusters were observed in all 3 dogs, and when the effect of seizure clusters was decreased by considering the subset of seizures separated by at least 4 hours, the forecasting performance remained better than chance for a subset of algorithm parameters. These results demonstrate that seizures in canine epilepsy are not randomly occurring events, and highlight the feasibility of long-term seizure forecasting using iEEG monitoring.

## Introduction

Epilepsy is a common neurological disorder affecting 0.5–1% of the world's population [1], and according to the World Health Organization accounts for nearly 1% of the entire global burden of disease [2]. Pharmacotherapy with anti-epileptic drugs is the mainstay of epilepsy treatment, but 20–40% of patients continue to have seizures despite medications [3]. A significant, if not the most important, cause of epilepsy related disability for patients is the uncertainty of when seizures occur [4]–[6]. Even patients with infrequent seizures report persistent anxiety about when their next seizure will strike [6]. In addition, patients take medications daily that produce cognitive and physical side effects for events that may occur infrequently. The ability to forecast seizures would make individualized epilepsy treatment possible, and patients could be warned of their impending seizures and take medications only when needed to prevent seizures. Because of the potential clinical impact, seizure forecasting has stimulated intense interest [7].

Multiple lines of investigation support the hypothesis that ictogenesis, the process of seizure generation, is not random. The recurrent seizures that define focal epilepsy originate from a localized brain region, and in most patients are associated with a stereotypic electroencephalography (EEG) discharge with characteristic spectral pattern [8], [9]. The spatial reproducibility of seizure onsets is the basis of successful epilepsy surgery, where focal resection of the brain tissue generating seizures can cure epilepsy [10]. The stereotypical pattern of seizure onset recorded with intracranial EEG (iEEG) is critical for 1^st^ generation devices that use algorithms to detect focal seizures and deliver responsive stimulation to abort them [11]. In addition to the spatial and spectral reproducibility of seizures, multiple studies show that seizures tend to cluster in time [12], [13] and exhibit underlying periodicities [14], [15]. This spatiotemporal clustering of seizures suggests a fixed network generating seizures and the potential for spatial and temporal seizure forecasting. Successful seizure forecasting requires the existence of a pre-ictal state associated with an increased probability of seizure generation and a physiological signal that distinguishes the pre-ictal (ictogenic) state from the inter-ictal state [15]–[17]. Physiological evidence that spontaneous seizures arise from periods of increased seizure probability, i.e. a pre-ictal state, comes from multiple lines of investigation. Some of the earliest clinical descriptions of epilepsy reported on precursory symptoms extending for hours and even days prior to seizures [18]. Subsequent studies have shown that in some patients, self-reported prodromes forecast seizures better than chance [19]. Physiological changes reported to occur prior to seizures include changes in cerebral blood flow [20], [21], blood oxygenation [22], blood oxygen-level dependent signal [23], and cortical excitability [24], [25]. The vast majority of seizure forecasting investigations have used EEG for quantifying interictal and pre-ictal brain states(for reviews see Mormann et al. [16]; Andrzejak et al. [26]).

Seizure forecasting, however, has remained controversial. Many early studies investigating seizure forecasting with intracranial EEG (iEEG) were later demonstrated to be statistically flawed [16], [26]. However, when using appropriate statistical tests some patients continued to show evidence for a pre-ictal state [27]–[29]. In many patients seizures are relatively rare events, and large data sets with multiple seizures and associated long inter-ictal periods are needed to rigorously evaluate forecasting algorithms [16], [29], [30]. The surgical evaluation of drug resistant epilepsy using intracranial electrodes provides a unique opportunity to directly investigate the generation of spontaneous seizures. Unfortunately, iEEG data sets from patients undergoing evaluation for epilepsy surgery are of relatively short duration and compromised by the acute effect of surgery and relatively rapid drug tapers used to capture adequate seizures for localization within a short period of time. Most of these clinical recordings only span a few days to a week. The inability to record long-term continuous electrophysiology and spontaneous seizures has significantly hindered progress in seizure forecasting. A similar data limitation complicates other areas of science directed at forecasting rare events, such as earthquakes [31], that also require long recordings to get adequate number of events.

We previously described an implantable device capable of acquisition of high quality, continuous iEEG over many months in dogs with naturally occurring epilepsy [32], [33]. The same device has recently been used in a first-in-human trial in Australia [7], [29]. Canine epilepsy [34] appears to be a good model of human epilepsy with similar epidemiology, clinical features, electrophysiology [35], [36], and response to anti-epileptic drugs [37]. Approximately 65% of canine seizures are characterized as focal onset with or without secondary generalization [38], and approximately 25% of dogs are not controlled with medications [39]–[42].

Here we evaluate a computationally simple seizure forecasting algorithm based on iEEG spectral power in multiple bands. Three dogs with naturally occurring epilepsy were instrumented with an implanted device recording 16 channels of continuous iEEG. Spontaneously occurring seizures were automatically detected and visually verified to create accurate long-term seizure catalogs. Long-term, continuous, iEEG records (ranging from 6.5 to 15 months) containing multiple seizures (15–83 events) were evaluated. A seizure forecasting algorithm using multiple spectral iEEG power band features and a variable seizure warning time was demonstrated to forecast seizures significantly better than chance. The results support the feasibility of long-term seizure forecasting in naturally occurring canine epilepsy. The ability to forecast seizures in canine epilepsy will allow exploration of new therapeutic approaches to epilepsy, such as responsive therapy to prevent seizures before they occur.

## Subjects and Methods

All studies had prior approval of the University of Minnesota Institutional Animal Care and Use Committee where the animals are maintained.

### Subjects

Seven mixed hounds with naturally occurring epilepsy and spontaneous seizures were implanted with the NeuroVista Seizure Advisory System previously described [32], [33]. All dogs had normal neurological examinations and MRI. The dogs were housed in the University of Minnesota canine epilepsy monitoring unit and continuously monitored (24 hours/day) with video and iEEG. The dogs were maintained on anti-epileptic medications during this study. Three dogs had an adequate number of seizures and prolonged interictal recordings suitable for analysis (Table 1).

**Table 1:** Subject IDs, imaging results, and specifics of iEEG records for three canines with naturally occurring epilepsy implanted with the NeuroVista Seizure Advisory System.

Subject ID (Breed)	MRI Brain	Recording duration, days	Number all seizures	Number lead seizures
002	Normal	197*	27	27
Mixed				
004	Normal	330	15	8
Mixed				
007	Normal	451	83	18
Mixed				
Group totals (mean ± std)		978	125	53
		(326±127)	(41.7±36.3)	(17.7±9.5)

Lead seizures were defined as seizures preceded by at least 4 hours of non-seizure.

Full record was 475 days in duration; only final 197 days used for forecasting to avoid post-surgical non-stationarities in iEEG.

### Surgical protocol

**Figure 1:**
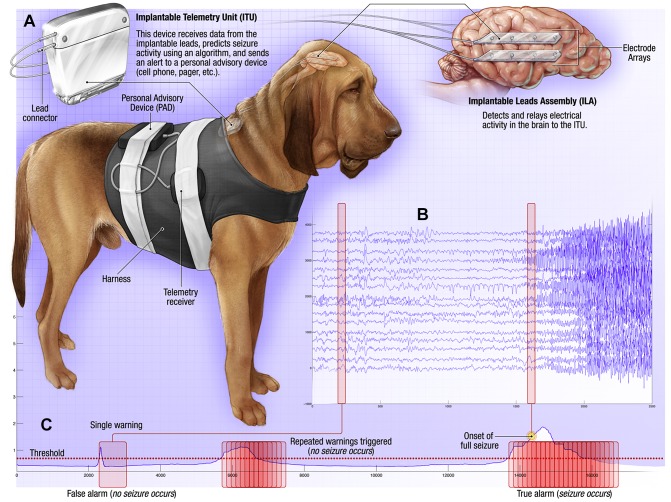
Seizure Advisory System (SAS) in Canines with Epilepsy. (A) The implantable device for recording and storing continuous iEEG includes: Implantable Lead Assembly (ILA) placed in the subdural space (right), Implantable Telemetry Unit (ITU), and Personal Advisory Device (PAD). The system acquires 16 channels of iEEG and wirelessly transmits the data to the PAD. Data is stored on a flash drive and uploaded weekly via internet to a central data storage site. (B) Sixteen channels of intracranial EEG recorded with SAS. A focal onset, secondarily generalized seizure is shown. The top 1–8 channels are from the left hemisphere and 9–16 from the right hemisphere, as shown on the brain schematic above. The onset of the seizure is from left hemisphere (underlined) electrodes 3 & 4. (C) Schematic of temporal profile of forecast seizure probability and threshold defining the pre-ictal state, i.e. period of increased seizure probability. When the forecast probability exceeds the defined threshold a fixed duration warning is triggered. Consecutive warnings that occur within the duration of a prior warning are combined, allowing for variable duration warnings. i) Single warning triggered without seizure (false positive warning). ii) Multiple consecutive warnings combined into prolonged warning without seizure (false positive warning). iii) Compounded warning prior to seizure onset (true positive seizure warning prior to electrographic seizure onset).

As described previously [32], dogs were anesthetized using a standardized protocol for intracranial surgery. Bilateral craniectomies were performed using standard aseptic procedures and two silicone strip electrodes each containing 4 contacts were placed bilaterally in the subdural space (Figure 1A). Lead tails were tunneled subcutaneously to the telemetry unit implanted in a dorsal tissue pocket on the dog.

### Device (Figure 1A)

A custom implantable iEEG acquisition system was used to acquire long-term continuous iEEG in three dogs [32], [33]. The system has three major components: (1) Implantable Lead Assembly (ILA) consisting of four silicone strips each containing four platinum-iridium contacts (4 mm^2^ surface area) separated by 20 mm; (2) Implantable Telemetry Unit (ITU); and (3) external Personal Advisory Device (PAD). The iEEG signals are recorded from the ILA contacts, filtered, amplified, and digitized (sampling rate 400 Hz) within the ITU and then wirelessly transmitted to the external PAD device. The ITU is charged daily for approximately 1 hour via an external battery powered device. The PAD was kept on the dog's back within a harness, and collected continuous iEEG data wirelessly from the ITU. The PAD has embedded seizure detection algorithms, and includes a user interface with functional lights for seizure warning, audible alarms, text messaging, and email algorithm outputs [33].

*Data curation:* For each dog 16-channel iEEG recordings (Figure 1B) were wirelessly transmitted to the PAD and stored on a flash memory card. Continuous video and iEEG data were archived to a central storage each week. A high sensitivity automated seizure detection algorithm was used to detect candidate seizures [43]. All candidate detections were visually reviewed and correlated with the continuous video to verify clinical seizure activity. The entire iEEG record was annotated by expert readers and all subclinical and clinical seizures verified. Canine data from this study are freely available on the iEEG portal (https://www.ieeg.org/).

### Feature extraction

Seizure forecasting requires a physiological signal that is characteristic of the hypothesized pre-ictal state. Here we investigated the spectral power of multichannel iEEG as a source of candidate features to distinguish the pre-ictal and inter-ictal states. Average reference iEEG was used for computation. In each of the 16 iEEG channels, the iEEG record (sampling rate 400 Hz) was partitioned into non-overlapping 1-minute blocks, each block Fourier transformed, and the resulting power spectrum (0.1–200 Hz) divided into 6 frequency bands: delta (0.1–4 Hz), theta (4–8 Hz), alpha (8–12 Hz), beta (12–30 Hz), low-gamma (30–70 Hz), and high-gamma (70–180 Hz). Within each frequency band the power was summed over band frequencies to produce a *power-in-band* (PIB) feature. These features were aggregated into a feature vector containing 96 PIB values (16 channels×6 bands) (Figures 2.1, 2.2, 2.3). The resulting feature vectors, one per 1-minute block in the original record, were collected over both pre-ictal and inter-ictal periods to form the training and testing data sets for the algorithm.

**Figure 2:**
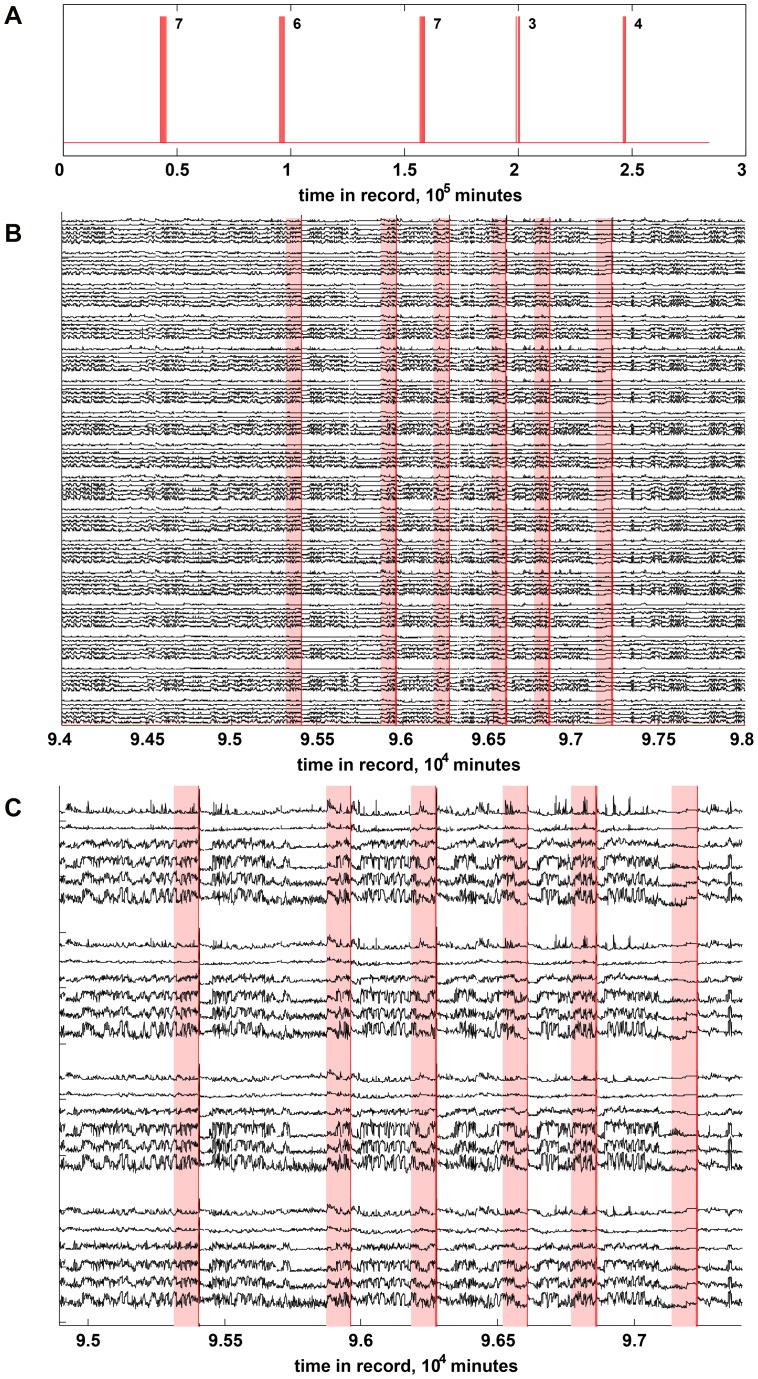
Temporal profile of seizures. (A) Full temporal record for canine 002, showing time of occurrence of the 27 clinically verified seizures (vertical red lines). The seizures fall into 5 clusters; each cluster is annotated with the number of seizures in the cluster. (B) Temporal profile of 96 power-in-band (PIB) features for canine 002, spanning approximately 2.8 days in the vicinity of seizure cluster 2 in (A). Each grouping of 6 traces shows the 6 PIB features derived from one iEEG recording channel; from bottom to top these features capture the power-in-band of the delta (δ: 0.1–4 Hz), theta (θ: 4–8 Hz), alpha (α: 8–12 Hz), beta (β: 12–30 Hz), gamma-low (γ-low: 30–70 Hz) and gamma-high (γ–high: 70–180 Hz) frequency bands, respectively. The grouping for channel 1 is at the bottom of the figure, and channel 16 at the top. Vertical red lines locate the occurrence of individual seizures. Light red shading indicates the 90-minute period preceding each seizure, which was labeled as pre-ictal for training purposes; everything else (white background) was treated as the inter-ictal state. (C) Expansion of traces for channels 9–12 in (B.

### Classifier training and testing

Logistic regression classifiers were trained to discriminate labeled pre-ictal and inter-ictal blocks using combinations of PIB features. For training purposes, blocks between 90 minutes preceding a seizure and the seizure itself were given a pre-ictal label, and all other blocks were labeled as inter-ictal. When applied to test data, the output of the trained classifier was a relative seizure risk for each test block on a continuous scale between 0 and 1 (Figures 3.1, 3.2). Classifier performance was assessed via 10-fold cross-validation, where folds were formed by dividing the entire record into 10 contiguous sub-records.

**Figure 3:**
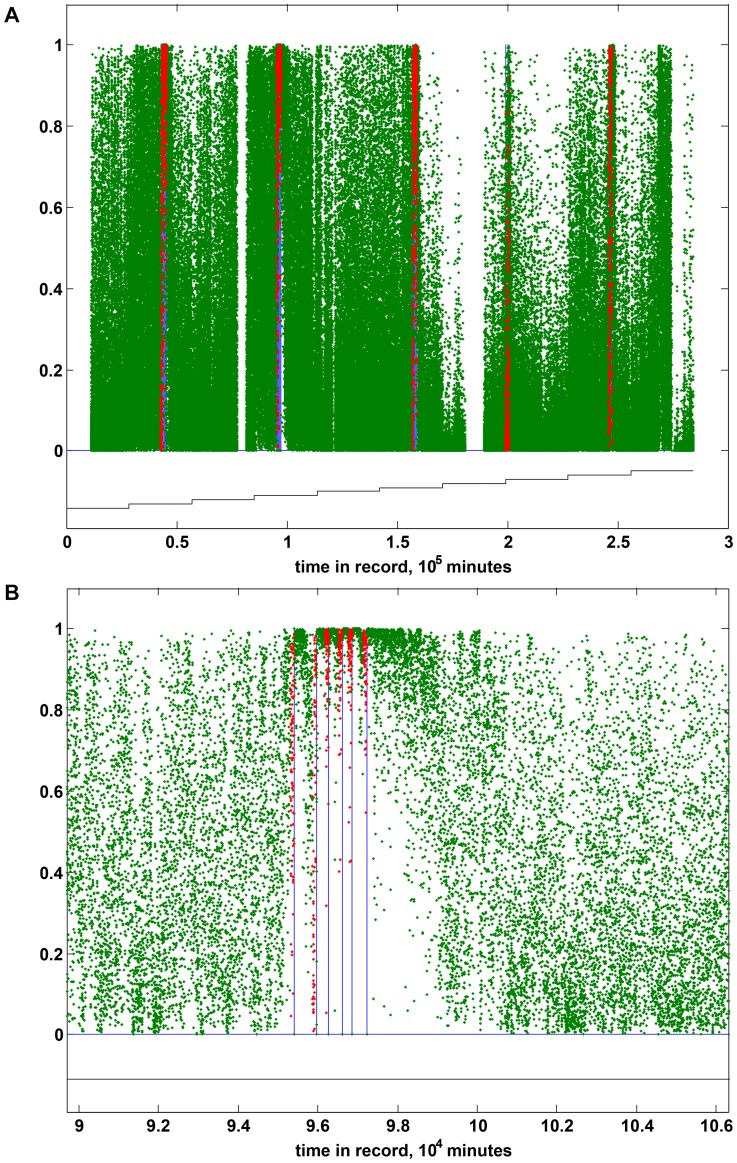
Seizure probability versus time. (A) The seizure probability for 1-minute blocks of full iEEG record of canine 002, as predicted by a logistic regression classifier trained on 96 power-in-band (PIB) features. Green and red dots indicate blocks labeled inter-ictal and pre-ictal, respectively, for classifier training, and vertical blue lines indicate occurrences of clinically verified seizures. Gaps in the plot correspond to periods of invalid data. The staircase underneath the plot delineates the 10 cross-validation partitions used during classifier training and testing. (B) Seizure probabilities for 1-minute blocks of iEEG record of canine 002 in vicinity of seizure cluster 2 (second from left in Figure 3 (A)), as predicted by a logistic regression classifier trained on 96 power-in-band (PIB) features. Green and red dots indicate blocks labeled inter-ictal and pre-ictal, respectively, for classifier training, and vertical blue lines indicate occurrences of clinically verified seizures.

### Forecasting algorithm

The seizure forecasting algorithm triggers a seizure warning when the classifier output exceeds a defined seizure risk threshold. The threshold is chosen adaptively so that total time in warning approximately matches a predefined target time in warning. Once triggered, the warning persists for a defined period of 90 minutes. Additional warnings that begin prior to termination of the previous warning are combined with it into a single variable duration warning period (Figure 1C). The variable duration of warning prior to a seizure encompasses the concept that the pre-ictal state is a period of increased seizure probability, and that seizures may occur at any time during the warning period [27], [29]. To distinguish seizure forecasting from simple seizure detection, a 5 minute forecast horizon is used. The beginning of the warning period must precede the seizure by the forecast horizon for it to be a valid forecast. That is, seizures not preceded by a warning beginning at least 5 minutes prior to seizure onset are false negative forecasts. If a seizure does not occur at some point during the warning period the warning is counted as a false positive forecast.

### Statistical analysis

Performance of the seizure forecasting algorithm was statistically evaluated using a Poisson-process chance prediction algorithm, as described previously by Snyder et al. [27]. The Poisson-process algorithm generates chance predictions for direct comparison to a candidate seizure forecasting algorithm. The Poisson-process algorithm uses the same parameters, including warning duration time, warning persistence rules, and forecasting horizon, as the candidate algorithm, and is constrained to have same total time in warning to control for specificity. A candidate algorithm must perform significantly (p<0.05) better than a matched chance predictor in order to claim evidence for seizure forecasting [44], [27].

## Results

In three dogs, continuous, long-term iEEG recordings were obtained and all clinical and subclinical seizure activity annotated. Table 1 shows the recording duration and number of seizures. The average duration of recordings was 326±127 days with a total of 125 seizures (41.7±36.3). Seizures separated by at least 4 hours from any preceding seizure were labeled lead seizures.

A simple forecasting algorithm using multiple spectral power bands as features to classify inter-ictal and pre-ictal data segments was tested on the three large datasets. The forecasting sensitivity, duration of recording spent in warning, number of false positive per day, and p-values are shown in Table 2. With the forecasting horizon set to 5 minutes, and triggered seizure warning duration of 90 minutes, forecasting runs with a range of different target total time in warning (0.1 to 0.5 of record) were evaluated (Table 2). The algorithm demonstrated seizure forecasting better than chance (p<0.05) for a range of total warning times for all 3 dogs when considering all seizures. Seizure clusters were observed in all 3 dogs (e.g. Figure 2.1). If the effect of clusters was decreased by considering only lead seizures, the forecasting performance remained better than chance for a subset of algorithm parameters. Dog 002 in particular showed very good seizure forecasting (74% of seizures forecasted, average false positives (FP) 2.8/day, p<0.00005, at total warning time = 0.3). Higher sensitivity was achieved at the expense of additional FP (e.g. 90% of seizures forecasted, average FP 3.0/day, and p<0.00005 at total warning time = 0.4). For Dog 004 seizure forecasting was better than chance when considering all seizures (73% of seizures forecasted, average FP 2.0/day, and p<0.0007 at total warning time = 0.3), but when considering only lead seizures, the performance did not reach significance. For Dog 007 seizure forecasting was significantly better than chance for both all seizures and lead seizures only, at total warning time = 0.3 (89% and 56% of seizures forecasted, with p<0.00005 and p<0.05 respectively; average FP 1.4/day).

**Table 2:** Seizure forecasting results for three canines with naturally occurring epilepsy implanted with the NeuroVista Seizure Advisory System.

ID	TIW	S_n_	*p*	S_n-lead_	*p*_n-lead_	False Positive/day
002	0.1	0.482	**0.0000**	0.482	**0.0000**	1.293
002	0.15	0.593	**0.0000**	0.593	**0.0000**	1.818
002	0.2	0.667	**0.0000**	0.667	**0.0000**	2.257
002	0.3	0.741	**0.0000**	0.741	**0.0000**	2.792
002	0.35	0.741	**0.0001**	0.741	**0.0001**	2.910
002	0.4	0.889	**0.0000**	0.889	**0.0000**	3.074
002	0.5	0.889	**0.0001**	0.889	**0.0001**	3.186
004	0.1	0.000	0.2435	0.000	0.6081	0.811
004	0.15	0.133	1.0000	0.125	1.0000	0.794
004	0.2	0.467	**0.0141**	0.250	1.0000	1.079
004	0.3	0.733	**0.0007**	0.500	0.2534	1.954
004	0.35	0.733	**0.0035**	0.500	0.4691	2.335
004	0.4	0.733	**0.0183**	0.500	0.7290	2.670
004	0.5	0.800	0.0700	0.625	0.7407	3.026
007	0.1	0.759	**0.0000**	0.222	0.7548	0.658
007	0.15	0.819	**0.0000**	0.278	0.5582	0.791
007	0.2	0.843	**0.0000**	0.389	0.1658	0.991
007	0.3	0.892	**0.0000**	0.556	**0.0421**	1.427
007	0.35	0.892	**0.0000**	0.556	0.0895	1.617
007	0.4	0.904	**0.0000**	0.556	0.2270	1.695
007	0.5	0.916	**0.0000**	0.611	0.2459	1.927

A logistic regression classifier was trained and used to predict iEEG data (1 minute blocks) as either pre-ictal or inter-ictal, using power-in-band (PIB) features. Each classifier used 10 of 96 available PIB features, chosen during the training process via forward selection. Forecasts and subsequent statistical analyses were based on different target proportions of total time in warning, ranging from 0.1 to 0.5. A 10-fold cross-validation scheme was used for all phases of feature selection, classifier training, seizure forecasting, and statistical analysis. For each dog a range of values for time in warning (**TIW**) are considered. The Sensitivity (**S_n_**) and p-value (***p***) are reported separately for all seizures and for lead seizures only (**S_n-lead_**, ***p*** **_n-lead_**) and have been adjusted to account for the performance of the chance prediction algorithm.

## Discussion

In 3 canines with naturally occurring epilepsy we investigated the feasibility of long-term seizure forecasting using iEEG. The ability to record, annotate, curate, and analyze large iEEG data sets spanning multiple months was demonstrated. In each of the three dogs studied, the results demonstrate seizure forecasting significantly better than would be expected by chance (using a generic Poisson-process algorithm) when considering all seizures. The analysis was performed on a massive high fidelity data set of 978 days of iEEG containing 125 recorded seizures. The large volume of interictal data and seizures made it possible to rigorously validate a simple seizure forecasting algorithm utilizing iEEG spectral power to classify brain states, and differentiate inter-ictal from pre-ictal states. A key component of the algorithm is the use of variable duration seizure warnings, rather than rigidly defining the duration of pre-ictal states [27]. The variable duration warning results from a simple technique that consolidates multiple overlapping warnings into a single longer duration warning (Figure 1C). The seizure forecasting algorithm performance was rigorously tested by benchmarking the performance against a chance prediction model, constrained to have an equal proportion of time spent in warning [27], [44]. Even when restricting the analysis to include only seizures separated by at least 4 hours (lead seizures), which diminishes statistical power by reducing the number of seizures from 125 to 53, seizure forecasting at levels better than chance was demonstrated in 2 of the 3 dogs.

As recently reviewed by Mormann, et al [16], previous studies have often failed to adequately evaluate the performance of proposed algorithms. Significant limitations of previous studies were the small data sets with few seizures and limited interictal iEEG. In the current study care was taken to avoid common pitfalls [16]: 1) The forecasting algorithm was tested on, continuous long-term recordings spanning multiple months. The long records contain adequate inter-ictal data and seizure numbers to definitively evaluate forecasting performance. 2) The sensitivity and specificity were rigorously tested and directly compared to a chance prediction algorithm constrained to have the same persistence parameters, forecasting horizon, and total time in warning. 3) Algorithm parameters were selected prior to analysis, and utilized across all animals. 4) The forecasting algorithm was optimized on training data (in-sample), but algorithm performance was calculated only on independent data (out-of-sample).

The results from three dogs demonstrate seizure forecasting performance at levels significantly better than chance when considering all seizures recorded (Table 2). The results show that canine epileptic seizures are not random events, and support the feasibility of seizure forecasting. The optimized level of performance for each dog (Table 2) supports the claim that clinically relevant seizure forecasting may be possible in humans, but highlights the need for long continuous iEEG recordings to capture an adequate number of seizures.

Seizure forecasting performance can likely be improved in the future. For example, the optimal recording bandwidth for differentiating interictal and pre-ictal states is not known. The current device has a relatively narrow bandwidth (sampling rate 400 Hz), but recent research has shown that the dynamic range of EEG activity generated from human brain spans a much wider spectrum, DC – 1000 Hz [45]. In addition, in this study we did not know the seizure onset zone location. The electrodes were placed according to a standardized protocol, similar to the approach in the recent human trial [29]. Previous studies have shown, however, that the EEG activity within and around the seizure onset zone is different [46]. Electrodes in the seizure onset zone could potentially improve seizure forecasting performance, and this is an area of ongoing research.

How this technology will translate to human subjects with epilepsy was recently evaluated in a clinical trial with encouraging results [29]. The optimal parameters for a clinical application of seizure forecasting likely depends on the application. For example, if seizure forecasting is used to trigger a therapy that does not disturb normal brain function (say low dose drug or sub-threshold stimulation, as used in the Neuropace investigational device [11]), then the algorithm could be adjusted for high sensitivity and a low false negative rate. This would lead to more false positives and more time in warning, but if the approach is able to prevent seizures with low levels of therapy without side effects it would be useful. This approach could reduce the exposure to the cognitive and physical side effects of anti-epileptic medications and render the drugs more efficacious. More challenging is a device solely for seizure warning, i.e. providing patients with a warning about impending seizures in order to reduce the potential for injury, and limiting social anxieties associated with the persistent fear of not knowing when the next seizure will strike. In this scenario, patients would likely demand a very high sensitivity (low false negative rate), because any missed seizure would be very disruptive. The device could prove dangerous if patients use the device to guide their ability to participate in certain activities, e.g. driving. Conversely, patients may not tolerate frequent false positive warnings as this would adversely affect their ability to participate in activities and could increase anxiety. Thus patients relying on a seizure forecasting device would likely have a low tolerance for missed seizures, i.e. false negatives, as well as frequent false positives. These possibilities are areas of current research.
